# Retained Intrarenal Guidewire Fragment After Endourological Stone Surgery: Antegrade Percutaneous Snare Retrieval and Literature Review

**DOI:** 10.3390/reports8030178

**Published:** 2025-09-15

**Authors:** Timoleon Giannakas, Aris Kaltsas, Ornella Moschovaki-Zeiger, Stavros Grigoriadis, Michael Chrisofos

**Affiliations:** 1 Third Department of Urology, Attikon University Hospital, School of Medicine, National and Kapodistrian University of Athens, 12462 Athens, Greece; tgiannakas@gmail.com (T.G.); ares-kaltsas@hotmail.com (A.K.); 2 Second Department of Radiology, Attikon University Hospital, School of Medicine, National and Kapodistrian University of Athens, 12462 Athens, Greece; m.z.ornella@gmail.com (O.M.-Z.); grigoriadis_stavros@hotmail.com (S.G.)

**Keywords:** intrarenal foreign body, guidewire fracture, hydrophilic guidewire, percutaneous retrieval, endovascular snare, retrograde intrarenal surgery, percutaneous nephrostomy, urosepsis, patient safety

## Abstract

**Background and Clinical Significance**: Retained intrarenal foreign bodies are rare adverse events after endourological stone surgery. Guidewire fracture or detachment is uncommon and can trigger infection, obstruction, or encrustation if unrecognized. We report antegrade percutaneous snare retrieval of a retained hydrophilic guidewire tip and provide a concise literature review (seven PubMed-indexed intrarenal cases identified by a structured search) to inform diagnosis, management, and prevention. We also clarify the clinical rationale for an antegrade versus retrograde approach and the sequencing of decompression, definitive stone management, and stenting in the context of sepsis. **Case Presentation**: A 75-year-old woman with diabetes presented with obstructive left pyelonephritis from ureteral and renal calculi. After urgent percutaneous nephrostomy, she underwent semirigid and flexible ureteroscopic lithotripsy with double-J stenting; the nephrostomy remained. During routine tube removal, the stent was inadvertently extracted. Seven days later she re-presented with fever and flank pain. KUB and non-contrast CT showed a linear 4 cm radiopaque foreign body in the left renal pelvis with dilatation. Under local anesthesia and fluoroscopy, a percutaneous tract was used to deploy a 35 mm gooseneck snare and retrieve the distal tip of a hydrophilic guidewire (Sensor/ZIP-type). Inflammatory markers were normalized; the nephrostomy was removed on day 5; six-week imaging confirmed complete clearance without complications. **Conclusions**: Retained guidewire fragments should be suspected in postoperative patients with unexplained urinary symptoms or infection. Cross-sectional imaging confirms the diagnosis, while minimally invasive extraction—preferably an antegrade percutaneous approach for rigid or coiled fragments—achieves prompt resolution. This case adds to the seven prior PubMed-indexed intrarenal reports identified in our review, bringing the total to eight, underscoring prevention through pre-/post-use instrument checks, immediate fluoroscopy when withdrawal resistance occurs, and structured device accounting to avoid “never events.”

## 1. Introduction and Clinical Significance

Intrarenal foreign bodies are uncommon in urological practice [1]. Historically, entry into the collecting system occurs through trauma or migration from adjacent organs. In contemporary practice, however, iatrogenic retention has emerged as a principal mechanism owing to the expansion of minimally invasive endourological procedures [1,2]. Instruments used in percutaneous and ureteroscopic surgery are generally robust, but fragmentation can occur, leaving components within the urinary tract [3]. Within this spectrum, retained intrarenal guidewire fragments are exceptionally rare and under-characterized, and current knowledge derives almost exclusively from isolated case reports and small series. In the largest early series of retained renal foreign bodies after endoscopic or percutaneous interventions (*n* = 21), only one was a guidewire remnant (≈5%), whereas ureteral stent and nephrostomy components predominated [4]. More recent literature continues to report only a handful of intrarenal guidewire events worldwide, underscoring the paucity of data and the need for practical guidance on recognition, retrieval strategy, and prevention [3,4].

Retained fragments may act as a nidus for encrustation and subsequent stone formation and can precipitate infection or obstructive complications [5,6]. Clinical presentations range from flank pain and hematuria to recurrent urinary tract infection, with severe cases progressing to hydronephrosis or urosepsis [6]. A variety of iatrogenic materials has been reported in the upper urinary tract, including double-J stent pieces, nephrostomy components, catheter or introducer fragments, and coiled or ruptured guidewires [6,7]. Early recognition and definitive removal using minimally invasive techniques are essential to avert long-term morbidity. Historically reported retrieval approaches include retrograde ureteroscopy with basket extraction or laser-assisted release for embedded tips, antegrade percutaneous techniques (including snare retrieval) or percutaneous nephrolithotomy, endoscopic combined intrarenal surgery (ECIRS), and—rarely—nephrectomy in non-functioning kidneys [8,9,10,11].

We herein report a fractured guidewire tip retained within the renal pelvis after endourological stone surgery, representing the eighth PubMed-indexed intrarenal case identified to date. It was managed with fluoroscopy-guided percutaneous snare retrieval, illustrating an effective minimally invasive solution to an unusual device-related complication. Given that retained floppy parts of guidewires and their retrieval have been previously documented, the present report emphasizes the clinical reasoning behind antegrade selection, staged sequencing in sepsis, and systems-level prevention, thereby adding practical procedural nuance to the literature.

## 2. Case Presentation

### 2.1. Patient Information

A 75-year-old woman with type 2 diabetes mellitus, chronic obstructive pulmonary disease, hypertension, and hypothyroidism was referred with obstructive left-sided pyelonephritis secondary to concurrent ureteric and renal calculi. Long-term medications included metformin 1000 mg twice daily, amlodipine 5 mg once daily, and levothyroxine 75 µg once daily. There were no known drug allergies, no history of prior urological instrumentation in the past 12 months, and no anticoagulant use. Baseline renal function was normal (estimated glomerular filtration rate ~65 mL/min/1.73 m^2^).

### 2.2. Timeline

At an outside center (Day 0), she underwent combined endourological stone surgery—percutaneous nephrolithotomy with adjunct ureteroscopic lithotripsy. A 6 Fr double-J ureteric stent was placed and a percutaneous nephrostomy (PCN) was left in situ. The operative note documented no resistance, device malfunction, or recognized guidewire damage. On postoperative day (POD) 5, the PCN was removed; during the same visit, the indwelling double-J stent was inadvertently extracted after accidental traction on the retrieval string. Seven days later (POD 12), the patient re-presented to the Emergency Department of Attikon University Hospital with fever and left flank pain and was admitted.

### 2.3. Clinical Findings at Emergency Department

On admission to our institution, temperature was 39.0 °C, heart rate 104 beats/min, and blood pressure 148/86 mmHg. The abdomen was soft; left costovertebral angle tenderness was marked, with no peritoneal signs. Urinalysis revealed pyuria and microscopic hematuria. Laboratory testing showed markedly elevated C-reactive protein with preserved renal function and a normal white-cell count (Table 1).

### 2.4. Diagnostic Assessment

**Figure 1:**
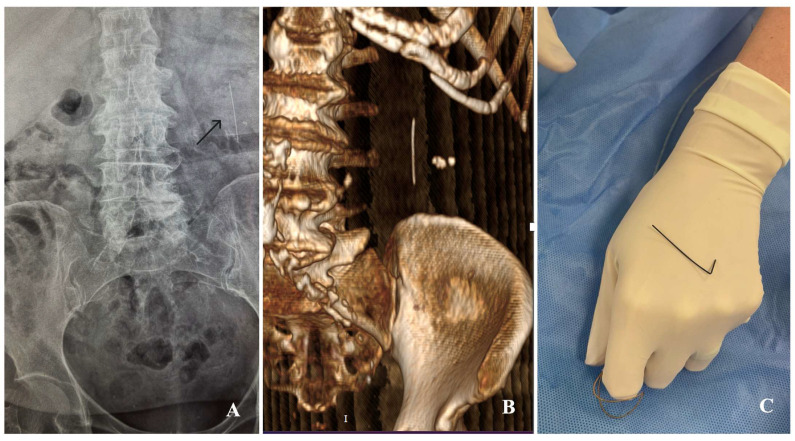
Stepwise documentation of the retained intrarenal guidewire fragment and its retrieval. (**A**) Supine KUB radiograph (plain abdominal X-ray) showing a linear radiopaque object in the left renal pelvis (arrow). (**B**) Volume-rendered non-contrast CT confirming the intrarenal location of the fragment. (**C**) Macroscopic view of the percutaneously retrieved hydrophilic guidewire tip (approximately 4 cm in length and 0.035-inch diameter) alongside the 35 mm snare loop used for its capture.

A kidneys–ureters–bladder radiograph demonstrated a linear radiopaque density projected over the left renal area. Non-contrast CT confirmed an approximately 4 cm linear metallic foreign body within the left renal pelvis with associated pelvicalyceal dilatation (Figure 1A,B). In the context of recent endourology, the appearance was most consistent with a retained guidewire fragment. Mid-stream urine culture yielded >10^5^ CFU/mL Escherichia coli susceptible to third-generation cephalosporins; paired blood cultures remained negative.

### 2.5. Therapeutic Interventions

Empiric intravenous piperacillin–tazobactam was initiated for obstructive urosepsis and later de-escalated to ceftriaxone when susceptibilities became available. Metformin was temporarily withheld during the acute septic episode, and venous thromboembolism prophylaxis was provided according to institutional protocol. Given ongoing sepsis with obstruction, an emergency PCN was placed to decompress the collecting system, with prompt clinical improvement. At this septic juncture, we deliberately avoided retrograde stenting or immediate ureteroscopy to prevent further manipulation of an infected, obstructed system, prioritizing drainage and source control.

Definitive removal of the foreign body was performed under local anesthesia and fluoroscopic guidance via the newly placed nephrostomy tract (posterior lower-pole calyx). An 8 Fr sheath was positioned, and a 35 mm gooseneck endovascular snare (Amplatz GooseNeck; Medtronic) was advanced to capture and retrieve the fragment (Figure 1C).

The extracted segment was identified as the distal hydrophilic tip of a 0.035-inch Sensor/ZIP-type guidewire (Boston Scientific). An 8 Fr nephrostomy catheter was re-inserted for short-term drainage. There were no intraprocedural complications; estimated blood loss was minimal and no transfusion was required.

After one additional week of clinical stabilization at Attikon—afebrile for ≥48 h, sterile urine culture, and down-trending inflammatory markers—definitive stone clearance was undertaken on POD 19. Semirigid ureteroscopic lithotripsy addressed a residual ureteric calculus, and flexible ureteroscopic laser lithotripsy treated residual intrarenal calculi. A double-J ureteric stent and the nephrostomy tube were left in situ at the end of the procedure to ensure drainage. This dual-drainage strategy was selected in the context of recent urosepsis to maintain robust decompression until sterility and unobstructed outflow were verified on follow-up imaging.

### 2.6. Outcomes and Follow-Up

Pyrexia resolved within 24 h of decompression and targeted antibiotics, and inflammatory indices normalized over 72 h. The nephrostomy catheter was removed on post-ureteroscopy day 5 following confirmatory imaging of unobstructed drainage. The double-J stent was removed uneventfully in clinic two weeks later. At six-week follow-up, ultrasound and low-dose CT showed no residual foreign material, no hydronephrosis, and satisfactory drainage. The patient remained asymptomatic at three months; serum creatinine was stable at 1.0 mg/dL and urine culture was sterile at follow-up.

### 2.7. Patient Perspective

At follow-up, the patient reported rapid relief of pain and anxiety once the cause had been identified and removed and expressed satisfaction with the minimally invasive approach. She noted reassurance after counseling regarding measures to prevent similar device-related events in future procedures. Written informed consent for publication of this case and the accompanying images was obtained.

## 3. Discussion

Retained intrarenal guidewire fragments are an exceedingly rare complication of endourological procedures. The incidence of retained foreign bodies in the renal collecting system after ureteroscopy is reported to be well below 1% [7], with most cases involving stent pieces, nephrostomy components, or laser fibers [12,13]. Because this rarity can lead to under-recognition, our report focuses not only on diagnosis and retrieval but also on the reasoning that supports approach selection and on concrete, system-level prevention steps.

We conducted a structured PubMed search (last updated 13 August 2025) using combinations of: “retained guidewire,” “guidewire fracture,” “intrarenal,” “renal pelvis,” “foreign body,” and “kidney.” Inclusion criteria were English-language reports explicitly documenting a guidewire (core, tip, or polymer coating) retained within the renal collecting system (pelvis/calyces), presented as case reports or embedded cases within series. We excluded ureter-only or bladder locations and non-indexed reports. This search identified six single-patient case reports describing intrarenal retained or fractured guidewires/coatings [1,3,4,7,10,11,14], and one additional intrarenal case within a series [4]; together with our patient, this yields eight intrarenal PubMed-indexed cases worldwide. For transparency, we separately note that including upper-tract/ureteric foreign bodies or non-indexed items slightly increases the overall number but does not change the intrarenal count [1,3,4,7,8,9,10,11].

A summary of these published cases, along with the present report, is provided in Table 2 to facilitate comparison in terms of patient characteristics, clinical presentation, retrieval approach, and outcomes.

Mechanical and manufacturing contributors to guidewire failure merit emphasis. Potential mechanisms of guidewire fracture include excessive torque, repeated reuse, manufacturing defects, or forceful withdrawal through tortuous or resistant tracts [11]. Hydrophilic-coated guidewires, while advantageous for navigating narrow or angulated passages, may be susceptible to polymer coating delamination or metal core fracture under certain conditions [15,16]. Case descriptions highlight sentinel intraoperative clues: resistance on withdrawal with subsequent mandrel protrusion and wire unwinding [3]; intraparenchymal coiling precipitated by an unfavorable needle-to-calyx angle and fibrosis [7]; and direct energy injury to the wire or its jacket from holmium:YAG laser [14]. These mechanisms create localized stress points (kinks, loops, or heat-weakened segments) that predispose to fracture, particularly at known weak interfaces such as the tip–shaft transition [3,7,14].

Importantly, device instructions for use (IFUs) from major manufacturers reinforce preventive measures that align with these failure modes. Hydrophilic wires should not be rotated or withdrawn against resistance; fluoroscopy should be used to determine the cause of hang-up; and the wire must be removed slowly without torque if the tip is trapped (Terumo GLIDEWIRE GT) [17]. Urological IFUs caution against laser contact with the wire and advise against reshaping or reusing damaged, bent, or kinked wires (Boston Scientific Sensor™/ZIPwire™); several advisories note that manipulation through sharp metal cannulas or needles can strip the polymer jacket, leaving retrievable fragments [18,19,20,21]. In practice, when resistance is encountered, stopping immediately, confirming position fluoroscopically, and—if needed—removing the entire assembly (scope, sheath, and wire) as a unit can mitigate the risk of shearing a segment [17,18,19,20,21]. Operator experience further mitigates risk: outcomes from high-volume endourologists indicate that disciplined handling reduces device injury and complications; this principle is echoed in the broader RIRS literature on learning and safety and supports rigorous technique and adherence to IFUs in daily practice [22].

Clinical manifestations range from asymptomatic incidental findings to flank pain, hematuria, recurrent urinary tract infections, or urosepsis [1]. Retained foreign material can act as a nidus for infection or encrustation, potentially leading to obstruction, hydronephrosis, or secondary stone formation. In this case, fever and flank pain prompted imaging, which revealed a 4 cm metallic fragment within the renal pelvis. Radiopaque materials such as guidewires are typically visible on plain radiographs, whereas radiolucent fragments require CT or MRI for detection. CT is the imaging modality of choice, enabling precise localization and assessment of associated complications [23].

Management aims at complete fragment removal with minimal morbidity. Selection of the retrieval approach depends on the size, configuration, encrustation, and location of the fragment. Retrograde ureteroscopy is generally sufficient for small, mobile fragments. By contrast, an antegrade percutaneous access offers a direct tract and wider working channel, which is advantageous for rigid, coiled, or embedded fragments [24]. In our case, a retrograde ureteroscopic approach was considered; however, given the fragment’s 4 cm length, stiff coiled configuration, and intrarenal location, an antegrade percutaneous route via the existing nephrostomy tract was deemed the safer and more effective option. Literature review indicates that most reported cases have been managed successfully with percutaneous removal, although retrograde approaches and, in rare circumstances, nephrectomy for non-functioning kidneys have also been described [9,11]. Percutaneous snare retrieval subsequently achieved complete removal without any parenchymal injury, further supporting the use of an antegrade approach in similar scenarios.

Preventive strategies are essential to avoid such complications. These include routine pre- and post-procedural inspection of all devices for integrity, careful monitoring for resistance during withdrawal, and immediate fluoroscopic evaluation if resistance is encountered [15].

Structured procedural checklists and meticulous device accounting further reduce the likelihood of retained instruments. From a patient-safety perspective, unintended retention of a foreign object after an invasive procedure is categorized as a “never event” by major safety bodies (e.g., NQF Serious Reportable Events; Joint Commission Sentinel Event Alerts), emphasizing mandatory disclosure, root-cause analysis, and systems-level prevention [25,26,27,28]. Briefly, a transparent discussion with the patient, prompt definitive management, and documentation of corrective actions are central to mitigating medico-legal exposure and preventing recurrences; institutional policies (including WHO Surgical Safety Checklist adaptations for endourology) should explicitly include guidewires as countable items [25,26,27,28,29]. Technical parameters of nephrostomy tract creation may also modulate complication risk; comparative studies and meta-analyses indicate that single-step (“one-shot”) dilation achieves bleeding and overall complication rates comparable to serial dilation while reducing operative and fluoroscopy time. Additionally, balloon dilation offers safety and efficacy comparable to Amplatz techniques, with some series reporting shorter dilation time and less blood loss [30,31,32].

Although the incidence of retained guidewire fragments is low, their potential for significant morbidity warrants heightened vigilance. Increased reporting of such cases contributes to improved recognition, facilitates refinement of preventive protocols, and may guide innovations in device design to reduce fracture risk. Early diagnosis through appropriate imaging and prompt minimally invasive retrieval, preferably by the antegrade route for rigid fragments, can lead to rapid resolution of symptoms and prevention of long-term sequelae.

## 4. Conclusions

Retention of intrarenal guidewire fragments constitutes a rare but clinically significant complication of endourological procedures. Early identification through appropriate imaging and timely minimally invasive removal—particularly via a percutaneous antegrade approach for rigid or embedded fragments—can prevent morbidity and obviate the need for open surgery. Adherence to strict instrument integrity checks, structured device accounting, and immediate fluoroscopic assessment when withdrawal resistance is encountered are pivotal preventive strategies. This case underscores the value of procedural vigilance and coordinated multidisciplinary management in achieving optimal patient outcomes. Meticulous instrument checks and prompt minimally invasive retrieval are crucial to avert serious complications from retained guidewire fragments.

## Figures and Tables

**Table 1:** Admission laboratory data.

Parameter	Patient Value	Reference Range *	Unit
C-reactive protein (CRP)	223	0–5	mg/L
Blood urea nitrogen (BUN)	18	7–20	mg/dL
Serum creatinine	1.1	0.5–1.1	mg/dL
Sodium	142	135–145	mmol/L
Potassium	3.8	3.5–5.1	mmol/L
Hemoglobin	12.5	12.0–15.5	g/dL
Platelets	230 × 10^3^	150–400 × 10^3^	/µL
White-blood-cell count	9.8 × 10^3^	4.0–11.0 × 10^3^	/µL
Urine leukocytes	58 (++++)	0–5	cells/HPF
Urine erythrocytes	31 (++)	0–5	cells/HPF

* Reference ranges are those of the Biochemistry and Microbiology Laboratories, Attikon University Hospital. CRP, C-reactive protein; BUN, blood urea nitrogen; HPF, high-power field; The semiquantitative notations “++++” and “++” correspond to standard dipstick grading for the degree of positivity in urine analysis, where “++++” indicates very high positivity and “++” indicates moderate positivity.

**Table 2:** Published case reports of retained intrarenal guidewire fragments, including patient demographics, clinical presentation, retrieval method, and outcomes, with comparison to the present case.

Author (Year)	Age/Sex	Clinical Presentation	Retrieval Method	Outcomes
Eisenberg et al., 2009 [4]	29/F	History of nephrolithiasis; retained inner core of guidewire 3 weeks after aborted PCNL	Percutaneous nephrolithotomy to remove residual stones and intact wire	Complete removal; uneventful recovery
Manassero et al., 2015 [7]	53/F	Flank pain and fever after prior staghorn stone surgery; CT: small kidney, residual stones, intrarenal “device” in lower pole	Open nephrectomy (anterolateral extraperitoneal)—guidewire fragment found tightly coiled and embedded in renal parenchyma	Uneventful recovery; discharged day six
Huang et al., 2015 [14]	46/M	Flank pain after ureteroscopy with Ho:YAG lithotripsy; imaging showed retained zebra guidewire tip in right renal pelvis and upper calyces	Flexible ureteroscopy via ureteral access sheath; grasped and removed tip under direct vision	Complete removal; discharged same day; no complications
Proietti et al., 2021 [10]	60/F	Lumbar pain and persistent urinary infection (*E. coli*); CT: long, tortuous calcified guidewire in right renal pelvis and lower calyx	Endoscopic combined intrarenal surgery (ECIRS) with complete removal of wire and residual stone fragments	Stone-free and wire-free at one month; no complications
Chen et al., 2021 [11]	40/F	Recurrent right flank pain, gross hematuria after PCND; prior urosepsis from upper ureteral stone	Retrograde intrarenal surgery with fURS, Holmium:YAG laser incision of parenchyma, basket retrieval	12 cm guidewire coating removed; symptom resolution; stent removed at three weeks; no complications
Smith, 2022 [3]	30/M	Recurrent upper-pole renal stones years after PCNL for UPJ calculus; retained 1.5 cm wire mandrel segment inside stone	PCNL for complete stone clearance; fragment removed	Stone-free; symptom resolution
Xiong et al., 2023 [1]	54/M	Flank pain; history of recurrent nephrolithiasis and prior PCNL; CT: C-shaped hyperdense object in inferior pole	PCNL with stone fragmentation and removal of guidewire tip	Uneventful; stent removed at one month
Present case (Giannakas et al., 2025)	75/F	Fever, flank pain, urosepsis	Percutaneous snare retrieval	Complete removal; asymptomatic at three months

Abbreviations: PCNL, percutaneous nephrolithotomy; CT, computed tomography; PCND, percutaneous nephrostomy drainage; URS, ureteroscopy; fURS, flexible ureteroscopy; UPJ, ureteropelvic junction.

## Data Availability

The original contributions presented in this study are included in the article. Further inquiries can be directed to the corresponding author.

## Abbreviations

The following abbreviations are used in this manuscript:

BUN	Blood urea nitrogen
CFU	Colony-forming unit
CRP	C-reactive protein
CT	Computed tomography
HPF	High-power field
KUB	Kidneys–ureters–bladder
PCN	Percutaneous nephrostomy

## References

[1] Xiong L., Kwan K.J.S., Hou J., Lu Z.Q., Wei G.G., Xu X. (2023). Incidental Finding of Intrarenal Foreign Guidewire During Percutaneous Nephrolithotomy: A Case Report and Literature Review. Am. J. Case Rep..

[2] Eswara J.R., Ko D.S. (2019). Minimally Invasive Techniques in Urology. Surg. Oncol. Clin. N. Am..

[3] Smith F.L. (2022). Perils of guide wire fracture—Unrecognized retained foreign body. Urol. Case Rep..

[4] Eisenberg M.L., Lee K.L., Stoller M.L. (2009). Endoscopic management of retained renal foreign bodies. Urology.

[5] Kiremit M.C., Koseoglu E., Acar O., Kilic M., Kordan Y., Canda A.E., Balbay M.D., Esen T. (2019). Distal ureteral stone formation over migrated Hem-o-lok clip after robot-assisted partial nephrectomy. Int. J. Surg. Case Rep..

[6] Alkan E., Basar M.M. (2014). Endourological treatment of foreign bodies in the urinary system. JSLS.

[7] Manassero F., Ortori S., Gabellieri C., Gabelloni M., Selli C. (2015). An unusual case of intrarenal coiled and ruptured guidewire. Arch. Ital. Urol. Androl..

[8] Kaplan A.G., Preminger G.M., Lipkin M.E. (2015). Combined Endoscopic and Percutaneous Retrieval of a Retained 4-Wire Ureteral Stone Basket. J. Endourol. Case Rep..

[9] Giusti G., Lisa A. (2018). Massive migration of embolization coils inside the renal pelvis. A rare complication that can be approached through percutaneous surgery. Cent. Eur. J. Urol..

[10] Proietti S., Rico L., Pavia M.P., Giusti G. (2021). Retained calcified guidewire in the kidney mimicking a renal stone. BMJ Case Rep..

[11] Chen B.H., Chang T.H., Chen M., Chen Y.H. (2021). Retrieval of intrarenal coiled and ruptured guidewire by retrograde intrarenal surgery: A case report and literature review. Open Med..

[12] Sahai A., Marsh H., Palmer J., Sriprasad S. (2009). Re: Eisenberg et al.: Endoscopic management of retained foreign bodies following flexible ureterorenoscopy for renal calculi (Urology 2009;73:1189–1194). Urology.

[13] McBroom S., Schenkman N.S., Stoller M.L. (2001). Retained laser fiber ureteral calculus. Urology.

[14] Huang Z., Fu F., Zhong Z., Xu R., Zhang L., Deng G., Zhao X. (2015). Zebra guidewire damage by Holmium: YAG laser and management of removal. Int. J. Clin. Exp. Med..

[15] Stawicki S.P., Evans D.C., Cipolla J., Seamon M.J., Lukaszczyk J.J., Prosciak M.P., Torigian D.A., Doraiswamy V.A., Yazzie N.P., Gunter O.L. (2009). Retained surgical foreign bodies: A comprehensive review of risks and preventive strategies. Scand. J. Surg..

[16] Symeonidis E.N., Symeonidis A., Anastasiadis A., Kaltsas A., Tsampoukas G., Mykoniatis I., Memmos D., Toutziaris C., Dimitriadis F., Vakalopoulos I. (2024). Breakage and detachment of the rigid cystoscope’s distal tip: An unusual case of urological instrument malfunction. Cent. Eur. J. Urol..

[17] Terumo Corporation GLIDEWIRE GT Guidewire—Instructions for Use (IFU). https://www.terumois.com/en-us/product-assets/glidewire-gt/GLIDEWIRE-GT-IFU.pdf.

[18] Boston Scientific Corporation ZIPwire Hydrophilic Guidewire—Prescriptive Information. https://www.bostonscientific.com/content/dam/bostonscientific/uro-wh/portfolio-group/prescriptive-information/URO-237312-AC_ZIPwire-Prescriptive-Information-FINAL.pdf.

[19] Boston Scientific Corporation Sensor Nitinol Wire with Hydrophilic Tip—Prescriptive Information (Brief Summary). https://www.bostonscientific.com/content/dam/bostonscientific/uro-wh/portfolio-group/guidewires/pdf/dfu/sensor-nitinol-wire-hydrophilic-tip-prescriptive-information.pdf.

[20] Boston Scientific Corporation Urological Guidewires—Prescriptive Information. https://www.bostonscientific.com/content/dam/bostonscientific/uro-wh/portfolio-group/guidewires/pdf/urological-guidewire-dfu.pdf.

[21] Cook Medical LLC UroStream Hydrophilic Wire Guide—Instructions for Use (IFU). https://ifu.cookmedical.com/data/IFU_PDF/T_UROS_REV3.PDF.

[22] Sforza S., Tuccio A., Grosso A.A., Crisci A., Cini C., Masieri L. (2020). Could surgical experience of adult endourologist overcome the learning curve of retrograde intrarenal surgery in children?. Urolithiasis.

[23] Mamoulakis C., Gorgoraptis P., Kehagias E., Karantanas A. (2018). Foreign body mimicking neoplasia of the renal pelvis on magnetic resonance imaging. Turk. J. Urol..

[24] Upadhyay S.P., Zahir M., Al Muttari H., Mallick P.N. (2015). A rare case of unusual migrated foreign bodies in kidney and their successful extraction using retrograde percutaneous nephrostomy. Qatar Med. J..

[25] Romano P., Gibbs V. Retained Surgical Items: Definition and Epidemiology. https://psnet.ahrq.gov/primer/retained-surgical-items-definition-and-epidemiology.

[26] National Quality Forum Serious Reportable Events in Healthcare—2011 Update: A Consensus Report. https://doh.wa.gov/sites/default/files/legacy/Documents/2900//NQF2011Update.pdf.

[27] The Joint Commission Sentinel Event Alert 51: Preventing Unintended Retained Foreign Objects. https://www.jointcommission.org/en-us/knowledge-library/newsletters/sentinel-event-alert/issue-51/.

[28] World Health Organization Implementation Manual WHO Surgical Safety Checklist 2009: Safe Surgery Saves Lives. https://apps.who.int/iris/handle/10665/44186.

[29] NHS England Never Events List 2018 (Updated February 2021). https://www.england.nhs.uk/wp-content/uploads/2020/11/2018-Never-Events-List-updated-February-2021.pdf.

[30] Akrivou D., Giannakodimos I., Adamos K., Kaltsas A., Mitakidi E., Karagiannis D., Chrisofos M., Skriapas K., Kratiras Z. (2025). Comparative Study of Mono J Single-Step Versus Two-Step Balloon Nephrostomy Placement for Urinary Tract Obstruction: Efficiency, Tolerability, and Complication Rates. J. Clin. Med..

[31] Peng P.X., Lai S.C., Ding Z.S., He Y.H., Zhou L.H., Wang X.M., Zhang G. (2019). One-shot dilation versus serial dilation technique for access in percutaneous nephrolithotomy: A systematic review and meta-analysis. BMJ Open.

[32] Peng P.X., Lai S.C., Seery S., He Y.H., Zhao H., Wang X.M., Zhang G. (2020). Balloon versus Amplatz for tract dilation in fluoroscopically guided percutaneous nephrolithotomy: A systematic review and meta-analysis. BMJ Open.

